# A rare duodenal perforation due to pancreatic stent migration

**DOI:** 10.1016/j.igie.2025.10.016

**Published:** 2025-10-24

**Authors:** Naoki Takemura, Akihiko Kida, Jun Asai, Tatsuya Yamashita, Eishiro Mizukoshi, Takeshi Urabe, Taro Yamashita

**Affiliations:** 1 Department of Gastroenterology, Public Central Hospital of Matto Ishikawa, Hakusan, Japan; 2 Department of Gastroenterology, Kanazawa University Hospital, Kanazawa, Japan; 3 Department of Gastroenterology, Saiseikai Kanazawa Hospital, Kanazawa, Japan; 4 Innovative Clinical Research Center, Kanazawa University Hospital, Kanazawa, Japan

A 74-year-old man presented with vomiting. Laboratory tests showed elevated liver enzyme levels and C-reactive protein levels, and computed tomography (CT) revealed common bile duct stones (CBDSs). The patient was diagnosed with acute cholangitis due to CBDSs. Endoscopic retrograde cholangiopancreatography (ERCP) with CO_2_ insufflation was planned for CBDS removal. Endoscopic retrograde cholangiography was successfully performed in the long-scope position using the pancreatic guidewire method. CBDSs were removed by an 8.7F stone extraction basket (VorticCatchV; Olympus Medical Systems, Tokyo, Japan) following endoscopic sphincterotomy and endoscopic papillary balloon dilation (StoneMasterV; Olympus Medical Systems). To prevent post-ERCP pancreatitis and cholangitis, a 5F × 3-cm plastic pancreatic stent (pPS) with no internal flap (Geenen Pancreatic Stent Set with No Internal Flap; Cook, Winston-Salem, NC, USA) and a 7F endoscopic nasobiliary drainage tube were placed. However, the patient self-extracted the endoscopic nasobiliary drainage tube on the same day as ERCP. Three days later, the patient had melena with mild abdominal pain. We suspected delayed hemorrhage after endoscopic sphincterotomy and planned endoscopic hemostasis using CO_2_ insufflation. Endoscopy revealed that a pPS had nearly completely migrated distally and penetrated the duodenal wall contralateral to the papilla of Vater (Figs. 1 and 2). After pPS removal by grasping forceps (FG-7L-1; Olympus Medical Systems), a pinhole-shaped perforation was found (Fig. 3), for which closure by endoscopic clipping (EC) (SureClip; Micro-Tech Co Ltd, Nanjing, China) was successfully performed (Fig. 4). CT was performed immediately, revealing free air on the liver surface and in the retroperitoneum. The patient was managed conservatively. Follow-up CT on day 14 post-EC showed resolution of free air, and the patient was discharged on day 18 post-EC.

**Figure 1:**
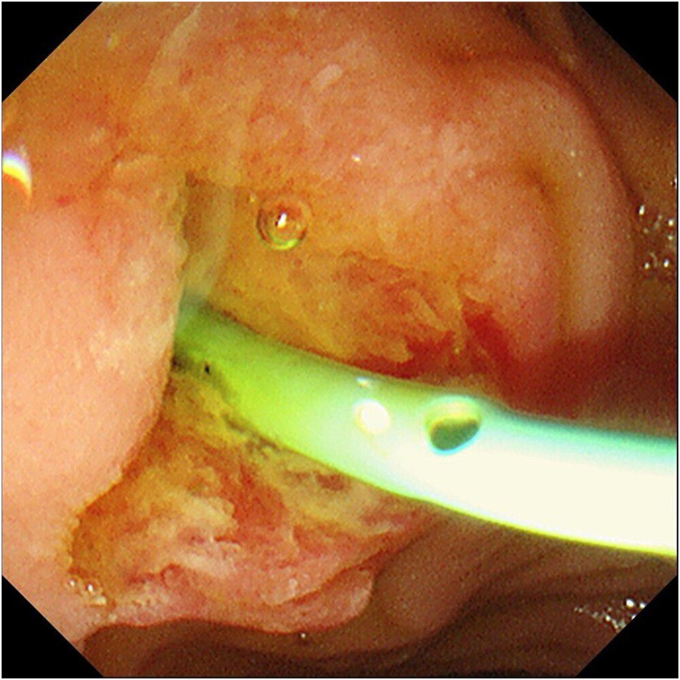
Endoscopy revealed that a plastic pancreatic stent had nearly completely migrated out of the pancreatic duct.

**Figure 2:**
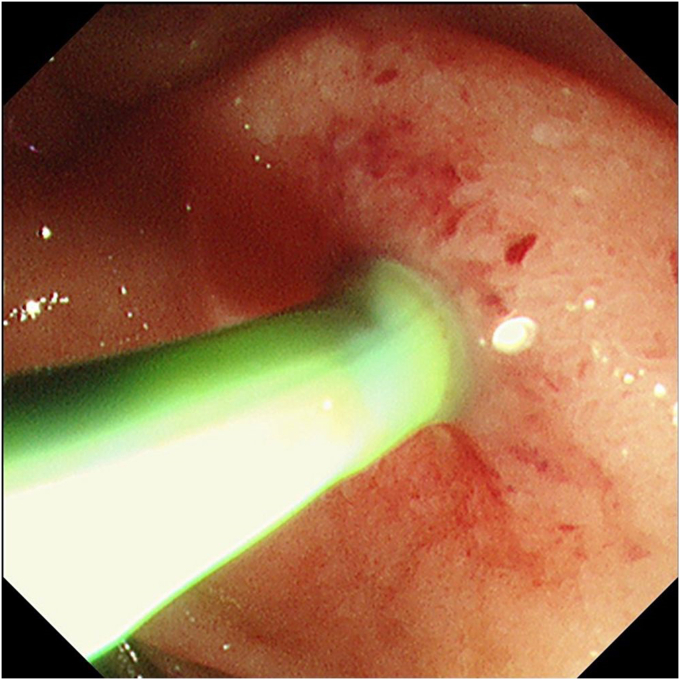
A plastic pancreatic stent penetrated the duodenal wall contralateral to the papilla of Vater.

**Figure 3:**
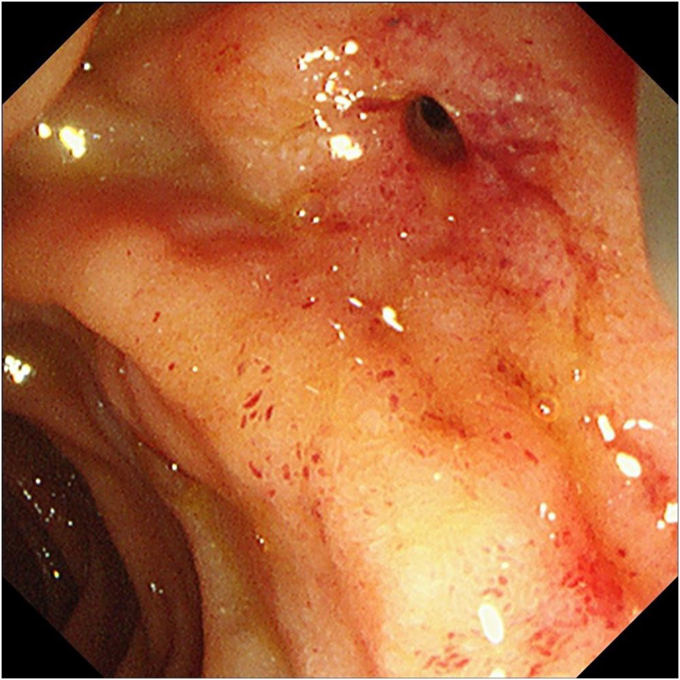
After plastic pancreatic stent removal by grasping forceps, a pinhole-shaped perforation was observed in the duodenal mucosa.

**Figure 4:**
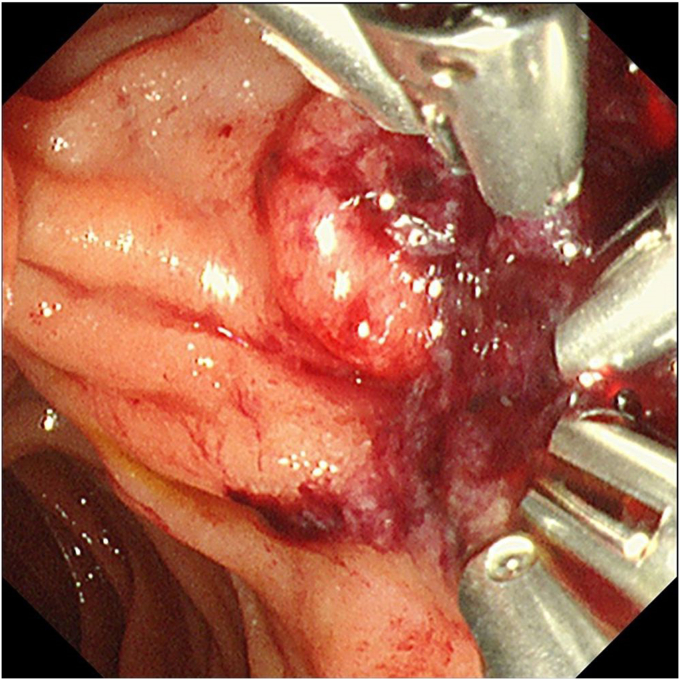
Closure by endoscopic clipping for a pinhole-shaped perforation was successfully performed.

Gastrointestinal perforations due to biliary stent migration are sometimes reported.^1^ Among such cases, the frequency of gastrointestinal perforations caused by plastic biliary stents are very high (87.1%).^2^ Therefore, duodenal perforations may occur because of pPS migration; however, reports of such cases are rare. The rare adverse event reported here may have occurred because of the extra force exerted on the pPS during self-extraction of the endoscopic nasobiliary drainage tube, causing the pPS to impact the contralateral duodenal wall with force. Although pPS placement to prevent post-ERCP pancreatitis is generally expected to result in spontaneous and safe stent dislodgement, especially when using stents without an internal flap, clinicians should be aware that a pPS may cause duodenal perforation.

## Ethical statements

This case was conducted in accordance with the ethical standards described in the latest revision of the Declaration of Helsinki.

Informed consent for patient participation and publication was received from the patient.

## DISCLOSURE

The author disclosed no financial relationships.
